# *In situ* identification of polyhydroxyalkanoate (PHA)-accumulating microorganisms in mixed microbial cultures under feast/famine conditions

**DOI:** 10.1038/s41598-020-60727-7

**Published:** 2020-02-28

**Authors:** Donhatai Sruamsiri, Parinda Thayanukul, Benjaporn Boonchayaanant Suwannasilp

**Affiliations:** 1 Department of Environmental Engineering, Faculty of Engineering, Chulalongkorn University, Bangkok, Thailand; 2 Department of Environmental Engineering, Faculty of Engineering, King Mongkut’s University of Technology Thonburi, Bangkok, Thailand; 3 Center of Excellence on Hazardous Substance Management, Chulalongkorn University, Bangkok, Thailand; 4 Department of Biology, Faculty of Science, Mahidol University, Bangkok, 10400 Thailand; 5 Research Network of NANOTEC-CU (RNN), Bangkok, Thailand; 6 Biotechnology for Wastewater Engineering Research Group, Chulalongkorn University, Bangkok, Thailand

**Keywords:** Environmental biotechnology, Environmental sciences

## Abstract

The accumulation of plastic waste in the environment has become a serious environmental problem worldwide. Biodegradable plastics, such as polyhydroxyalkanoate (PHA), could serve as green alternatives to petroleum-based plastics. In this study, a mixed microbial culture was enriched under feast/famine conditions using a sequencing batch reactor (SBR) with acetate as a carbon source. The enrichment could accumulate a maximum PHA concentration of 32.3% gPHA/g mixed liquor suspended solids (MLSS) in the 12^th^ cycle of SBR operation. The microbial community in this sludge sample was analyzed using 16 S rRNA gene amplicon sequencing (MiSeq). The results showed the dominance of Proteobacteria, represented by Alphaproteobacteria (13.26% of total sequences), Betaproteobacteria (51.37% of total sequences), and Gammaproteobacteria (23.44% of total sequences). Thauera (Betaproteobacteria) had the highest relative abundance, accounting for 48.88% of the total sequences. PHA-accumulating microorganisms in the enrichment were detected using fluorescence *in situ* hybridization (FISH) and a fluorescent dye, Nile blue A. Alphaproteobacteria and Betaproteobacteria were capable of accumulating PHA, while no Gammaproteobacteria were detected. Thauera spp. from Betaproteobacteria constituted 80.3% of the total PHA accumulating cells.

## Introduction

Massive generation of plastic waste has long been considered a worldwide environmental problem. The accumulation of plastic waste and microplastics in the environment can be detrimental to natural ecosystems. Polyhydroxyalkanoate (PHA) is a microbial polymer that can be used in biodegradable plastics, thereby offering a green alternative to nonbiodegradable, petroleum-based plastics. Currently, PHA can be commercially produced using pure cultures of bacteria, such as *Cupriavidus necator, Alcaligenes latus, Burkholderia cepacia*, and recombinant *Escherichia coli*^1,2^. However, the production costs are still high due to the required sterile processes.

Research on PHA production from organic wastewater using mixed microbial cultures has drawn great attention since it can reduce PHA production costs while applying the concept of resource recovery from wastewater. There are several approaches for the enrichment of PHA-accumulating microorganisms in mixed microbial cultures, including creating anaerobic/aerobic, microaerophilic/aerobic, or feast/famine conditions^3^. At present, feast/famine conditions can support the highest PHA content (89% gPHA/gMLSS) compared to the other approaches^4^.

The microbial community is an important factor that can affect PHA production in mixed microbial cultures. Previous research has investigated microbial communities in PHA production systems with mixed microbial cultures under feast/famine conditions. Various molecular techniques have been used, such as a 16 S rRNA gene clone library generation^5^, 16 S rRNA gene amplicon sequencing (MiSeq/HiSeq)^6,7^, polymerase chain reaction-denaturing gradient gel electrophoresis (PCR-DGGE)^8–11^, and fluorescence *in situ* hybridization (FISH)^9,10,12–17^. However, information on the microbial communities cannot explain which groups of microorganisms actually accumulate PHA in these systems and what roles those microorganisms play. Fortunately, PHA can be stained using fluorescent dyes such as Nile blue A. This technique, supported by FISH, enables us to identify the groups of microorganisms that accumulate PHA. Nevertheless, information on the groups of microorganisms that accumulate PHA and the conditions for PHA accumulation under a feast/famine feeding regime remains limited^4,9,10,12,13,16,17^. Considering that microbial communities can greatly vary in terms of substrates, operating conditions (e.g., solid retention time (SRT)), seed sludges, and environmental factors (e.g., pH and temperature)^13,18^, further information on microbial communities and PHA-accumulating microorganisms in mixed microbial cultures is still required. With the current advances in molecular techniques, more complete information on microbial communities can be achieved via next-generation sequencing, which, in combination with FISH and the fluorescence staining technique, can greatly assist in identifying PHA-accumulating microorganisms.

This study aimed to analyze the microbial community in a mixed-culture PHA-accumulating system under feast/famine feeding conditions using 16 S rRNA gene amplicon sequencing (MiSeq) and FISH in conjunction with the fluorescence-based PHA staining technique. The findings from this study improve our understanding of the roles of different groups of microorganisms in PHA accumulation in mixed microbial cultures. In addition, this study expands our knowledge of the microorganisms capable of accumulating PHA *in situ* in mixed microbial cultures, certain key microorganisms in which may not be individually cultivable.

## Materials and Methods

### Enrichment of PHA-accumulating microorganisms in an SBR

The seed sludge for the enrichment was taken from an aerobic sequencing batch reactor (SBR) in the wastewater treatment plant of a fruit juice-manufacturing factory. An SBR of 0.3 m × 0.3 m × 0.23 m (width × length × height) with a total wet volume of 20 L was used for the enrichment of PHA-accumulating microorganisms via a feast/famine feeding regime. The SBR was operated at room temperature (27.8 ± 0.83 °C) with an initial mixed liquor suspended solids (MLSS) of 3,000 mg/L. The SBR cycle consisted of five steps: (1) 5 min of synthetic wastewater feeding, (2) 47 h of aeration by air diffusers, (3) 5 min of sludge wastage, (4) 30 min of sludge settling, and (5) 20 min of decanting. The feast/famine conditions occurred in the second step, when aeration was provided. The SBR was operated at an SRT of 10 d. The synthetic wastewater consisted of acetate, 3,000 mg chemical oxygen demand (COD)/L; NH_4_Cl, 100 mg N/L; KH_2_PO_4_, 20 mg P/L; MgSO_4_, 500 mg/L; CaCl_2_, 10 mg/L; FeCl_3_, 10 mg/L; H_3_BO_3_, 4 mg/L; CuSO_4_·5H_2_O, 2 mg/L; MnCl_2_·2H_2_O, 0.3 mg/L; NaMoO_4_·2H_2_O, 2 mg/L; ZnSO_4_·7H_2_O, 2 mg/L; CoCl_2_·6H_2_O, 8 mg/L; NiCl_2_·6H_2_O, 2 mg/L; NaHCO_3_, 50 mg/L, as a pH buffer; and thiourea, 20 mg/L, as a nitrification inhibitor^6^. The pH was maintained in the 6.5–9.5 range using an automatic pH controller (Alpha 190/200, Thermo Scientific, USA). The COD and MLSS were measured at the beginning and end of each cycle. The COD removal efficiencies (%), which reflect the performance of the SBR in terms of wastewater treatment efficiencies, were calculated from

$$\frac{{{\rm{COD}}}_{0}-{{\rm{COD}}}_{{\rm{e}}}}{{{\rm{COD}}}_{0}}\times 100,$$

where COD_0_ and COD_e_ are the COD concentrations (mg/L) at the beginning and end of the cycle, respectively. In addition, the COD, MLSS, and PHA levels were measured over time during the 4^th^, 8^th^, 12^th^, 15^th^, 22^nd^, and 25^th^ cycles. In these cycles, initial specific rates of substrate utilization (gCOD/gMLSS-h), yields of PHA (gPHA/gCOD), and COD removal efficiencies (%) were estimated. The initial specific rates of substrate utilization (gCOD/gMLSS-h) were estimated during the first 2 h of SBR operation from

$$\frac{1}{{{\rm{MLSS}}}_{0}}[\frac{{{\rm{COD}}}_{0}-{{\rm{COD}}}_{2{\rm{h}}}}{2}],$$

where COD_0_ and COD_2h_ are the COD levels (mg/L) at the beginning of the cycle and at 2 h, respectively, and MLSS_0_ is the MLSS (mg/L) at the beginning of the cycle. The yields of PHA (gPHA/gCOD) were calculated from

$$\frac{[(\frac{{ \% {\rm{PHA}}}_{{\rm{\max }}}}{100})\times {{\rm{MLSS}}}_{{\rm{maxPHA}}}]-[(\frac{{ \% {\rm{PHA}}}_{0}}{100})\times {{\rm{MLSS}}}_{0}]}{{{\rm{COD}}}_{0}-{{\rm{COD}}}_{\max {\rm{PHA}}}},$$

where %PHA_max_ and %PHA_0_ are the maximum PHA content (% gPHA/gMLSS) and the PHA content (% gPHA/gMLSS) at the beginning of the cycle, respectively; MLSS_maxPHA_ and MLSS_0_ are the MLSS values (mg/L) when the PHA content was at the maximum level and at the beginning of the cycle, respectively; and COD_maxPHA_ and COD_0_ are the COD levels (mg/L) when the PHA content was at the maximum level and at the beginning of the cycle, respectively.

### Analytical methods

For the PHA quantification, sludge samples were centrifuged at 8,000 rpm for 5 min and oven-dried at 80 °C for 24 h. The dried samples were added into a 1:1 (v/v) mixture of chloroform and methanol containing 3% sulfuric acid. These solutions were heated at 80 °C for 3 h for methyl esterification of PHA^19^. The resulting methyl esters of hydroxybutyrate (HB) and hydroxyvalerate (HV) were measured using gas chromatography with a flame ionization detector (Agilent Technologies) equipped with an HP-INNOWax capillary column (30 m × 0.25 mm id 0.25 µm, Agilent Technologies). Poly(3-hydroxybutyrate-co-3-hydroxyvalerate) containing 12 wt % of HV was used as the PHA standard (Sigma-Aldrich, Co.), and benzoic acid served as an internal standard^6^. The COD and MLSS values were measured using the closed reflux and gravimetric methods, respectively^20^. The automatic pH controller monitored the pH values.

### Microbial community analysis using 16 S rRNA gene amplicon sequencing

The sludge sample from the SBR cycle that achieved the maximum PHA content was further analyzed for its microbial community using 16 S rRNA gene amplicon sequencing. A DNA extraction kit (FastDNA® Spin Kit for Soil, MP Biomedicals, USA) was used. The v4 region of the 16S rRNA gene was amplified using polymerase chain reactions (PCRs) with the universal primers for bacteria and archaea: 515 F: 5′-GTGYCAGCMGCCGCGGTAA-3′ and 806 R: 5′-GGACTACHVGGGTWTCTAAT-3′^21^. The PCR conditions were as follows: an initial denaturation at 95 °C for 3 min; 20 cycles of 95 °C for 30 s, 55 °C for 30 s, and 72 °C for 30 s; and a final extension at 72 °C for 5 min. The PCR product was purified using the GF-1 AmbiClean Kit (Gel & PCR, Vivantis Technologies). The purified PCR product was indexed using the Nextera XT Index Kit with 8 cycles of the above PCR conditions. Next, the indexed 16 S rRNA gene amplicon was purified using AMPure XP beads (Beckman Coulter, USA), pooled, and diluted to a final loading concentration of 4 pM. The pooled sample was sequenced on an Illumina MiSeq Sequencer at Omics Sciences and Bioinformatics Center (Chulalongkorn University, Bangkok, Thailand). The quality of the sequences was checked using FASTQC software. Paired-end reads were combined using PEAR^22^. Assembled reads in which 90% of the bases had quality scores less than 30 or reads shorter than 200 bp were removed using FASTX-Toolkit. The UCHIME method was used to remove chimeric sequences by implementing vsearch1.1.1 with the uchime_ref option against the chimera-free Gold RDP database^23,24^. Operational taxonomic unit (OTU) picking was conducted using the *pick_open_reference_otus.py* command with the SortMeRNA method in QIIME 1.9.0. The Greengenes database was used for taxonomic assignments. The sequences that failed to match the references were clustered de novo using SUMACLUST. OTUs with less than 0.1% reads were removed.

### Investigation of PHA-accumulating microorganisms

FISH was performed with the PHA staining technique using methods from previous studies^4,25,26^, with some modifications. A sludge sample (1 mL) from the SBR cycle that achieved the maximum PHA content was collected (the same sample that was used for the 16S rRNA gene amplicon sequencing analysis). The pelleted sample was fixed with 4% paraformaldehyde (SCBT, USA) at 4 °C for 1.5 h or overnight. Equal volumes of phosphate-buffered saline (PBS; 137 mM NaCl, 8.1 mM Na_2_HPO_4_·7H_2_O, 2.68 mM KCl, 1.47 mM KH_2_PO_4_; pH 7.2) were used to wash the sludge twice, and the sample was stored at −20 °C in a 1:1 solution in PBS with 99.5% ethanol. The fixed sludge was spotted on MAS-coated glass slides (Matsunami Glass, Osaka, Japan), air-dried, and dehydrated in 50%, 80%, and 99.5% ethanol consecutively for 3 min each. Hybridization solutions (900 mM NaCl, 20 mM Tris/HCl (pH 7.4), 0.01% SDS) were prepared with different formamide concentrations as required for each oligonucleotide probe^25^. Hybridization solutions containing the different probes and competitors were applied to the slides. The final concentration of each probe in the hybridization buffer was 0.1 pmol/µL. Hybridization was performed at 46 °C for 2–4 h. Excess probe was removed with the appropriate washing buffer by heating at 48 °C for 15 min followed by rinsing with cool dH_2_O^25^. The dried biomass was dipped into 1% (w/v) Nile blue A (Sigma-Aldrich, Germany) at 55 °C for 15 min to stain the PHA (blue color) before washing with 8% (v/v) acetic acid solution for 1 min, according to Johnson *et al*.^4^. After air-drying, 8 μL of DAPI solution (1 μg/mL, blue) was spotted onto the sample for 15 min and washed off with dH_2_O to enumerate the total microorganisms.

The oligonucleotide probes were used to determine the PHA accumulation capacity of Alphaproteobacteria, Betaproteobacteria, Gammaproteobacteria and *Thauera* spp. Table 1 shows the experimental conditions. The probes were synthesized and labeled with Cy3 (red) by Macrogen, Korea. The nonlabeled competitors (BET42a and GAM42a) were used at equimolar concentrations with the target probes. Slow-Fade solution and a cover glass were applied to the dried biomass. Microscopic observation was performed using an Olympus Fluoview FV10i confocal laser scanning microscope (Olympus, Japan). The percentage of total specific bacterial groups (red) was enumerated versus the total number of microorganisms (blue) or PHA-accumulating organisms (yellow). Twenty images were taken for each probe-hybridized sample. Sludge samples without PHA from the initial cycle of this SBR and the nitrifying reactor were used as a negative control for PHA staining for FISH hybridization.

**Table 1:** Oligonucleotide probes used for FISH in this study.

Target Microorganism	Probe	Sequence (5′–3′)	FA (%)	Reference
Alphaproteobacteria	ALF1b	CGTTCGYTCTGAGCCAG	20	Manz *et al*.^38^
	ALF968	GGTAAGGTTCTGCGCGTT	20	Neef^39^
Betaproteobacteria	Bet42a	GCCTTCCCACTTCGTTT	35	Manz *et al*.^38^
	Bet42a competitor	GCCTTCCCACATCGTTT		
Gammaproteobacteria	Gam42a	GCCTTCCCACATCGTTT	35	Manz *et al*.^38^
	Gam42a competitor	GGTAAGGTTCTGCGCGTT		
*Thauera* spp.	THAU832	TGCATTGCTGCTCCGAAC	30	Loy *et al*.^35^, Ricardo *et al*.^36^

## Results and Discussion

### Enrichment of PHA-accumulating microorganisms in an SBR

**Figure 1:**
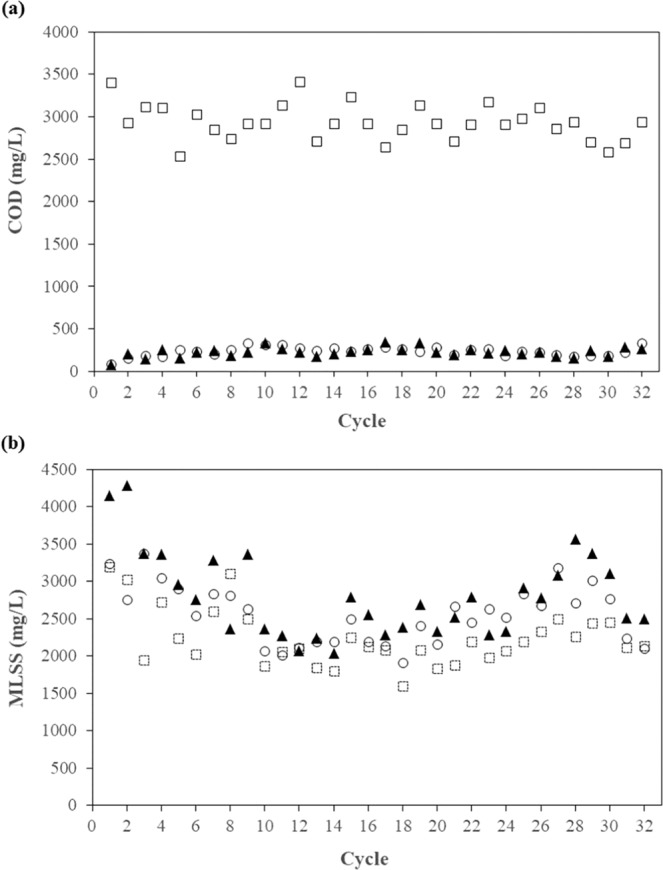
COD (**a**) and MLSS (**b**) levels in the SBR: □ at the beginning of the cycle (0 h); ▲ at the middle of the cycle (24 h); ○ at the end of the cycle (47 h).

PHA-accumulating microorganisms were enriched in the SBR under feast/famine feeding conditions. Figure 1a shows the COD levels in the SBR at the beginning (0 h), in the middle (24 h), and at the end (47 h) of the SBR cycles. The COD levels in the middle and at the end of the cycles were rather close, indicating that the SBR reached famine conditions during every cycle. Figure 1b shows the MLSS in the SBR. The MLSS increased in the middle of the cycles, suggesting biomass growth during the first half of the cycles. On the other hand, the MLSS decreased at the end of the cycles, suggesting biomass decay during the second half of the cycles, consistent with the famine conditions observed based on the COD levels. The average COD removal efficiency for all of the SBR cycles was 92.0 ± 1.9%, which was comparable to typical aerobic treatment processes, suggesting that this system can still effectively serve as a wastewater treatment system.

**Figure 2:**
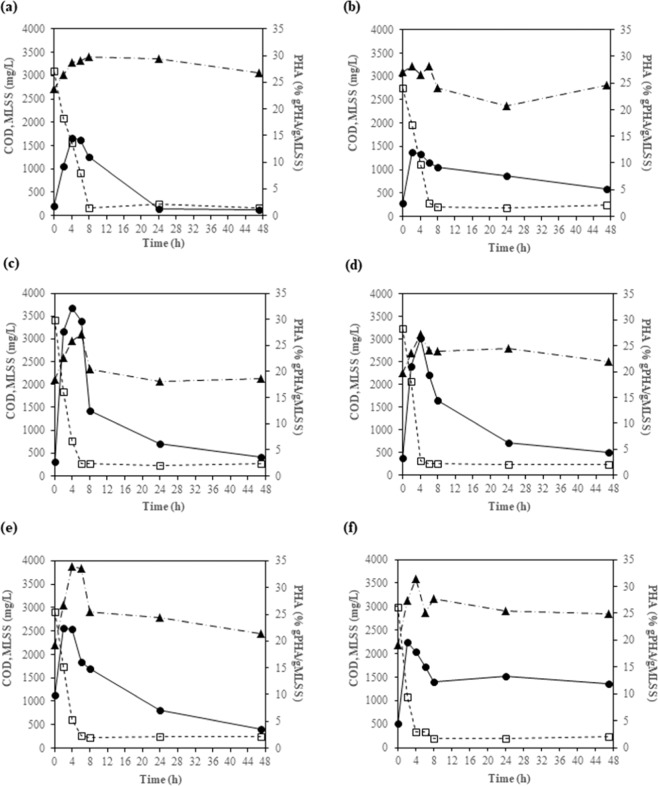
COD levels (□), MLSS values (▲), and PHA contents (●) in the 4^th^ (**a**), 8^th^ (**b**), 12^th^ (**c**), 15^th^ (**d**), 22^nd^ (**e**), and 25^th^ (**f**) cycles of SBR operation.

Figure 2 shows the COD and MLSS levels in the 4^th^, 8^th^, 12^th^, 15^th^, 22^nd^, and 25^th^ cycles in greater detail. According to the COD results, the lengths of the feast/famine conditions were approximately 6 h and 41 h, respectively, which corresponds to a feast/famine ratio of 0.146. This ratio is considered low, which is suitable for the enrichment of PHA-accumulating microorganisms^5,15^. In addition, Fig. 2 shows the PHA contents in the biomass in the 4^th^, 8^th^, 12^th^, 15^th^, 22^nd^, and 25^th^ cycles. In each cycle, the PHA content increased during the feast phase and reached a maximum at approximately 2–4 h. The PHA content gradually decreased during the famine phase, suggesting that PHA was used as the carbon source when the external substrate was depleted. Regarding the PHA composition, the PHA in all of the samples consisted of 100% HB. No HV was detected. This observation is consistent with previous studies that found 100% HB when acetate was used as a substrate for PHA production^4,27^.

The initial specific rates of substrate utilization (gCOD/gMLSS-h), maximum PHA contents (% gPHA/gMLSS), and yields of PHA (gPHA/gCOD) in the 4^th^, 8^th^, 12^th^, 15^th^, 22^nd^, and 25^th^ cycles are summarized in Table 2. The initial specific rates of substrate utilization appeared to be higher in the latter cycles (12^th^, 15^th^, 22^nd^, and 25^th^ cycles) than in the earlier cycles (4^th^ and 8^th^ cycles). Similarly, the maximum PHA contents in the latter cycles (12^th^, 15^th^, 22^nd^, and 25^th^ cycles) were higher than those in the earlier cycles (4^th^ and 8^th^ cycles). The higher initial specific rates of substrate utilization and the higher maximum PHA contents in the latter cycles were likely due to microbial selection and adaptation to the imposed feast/famine conditions. The average yield of PHA was 0.30 ± 0.06 gPHA/gCOD (~0.45 Cmol PHB/Cmol acetate), which was within the range 0.21–0.60 Cmol PHB/Cmol acetate previously observed by Johnson *et al*.^27^.

**Table 2:** Initial specific rates of substrate utilization (gCOD/gMLSS-h), maximum PHA contents (% gPHA/gMLSS), and yields of PHA (gPHA/gCOD) at different cycles of SBR operation.

Parameter	4^th^ cycle	8^th^ cycle	12^th^ cycle	15^th^ cycle	22^nd^ cycle	25^th^ cycle	Average ± SD
Initial specific rate of substrate utilization (gCOD/gMLSS-h)	0.19	0.13	0.37	0.26	0.27	0.43	0.27 ± 0.11
Maximum PHA content (% gPHA/gMLSS)	14.4	12.1	32.3	26.4	22.4	19.6	21.2 ± 7.5
Yield of PHA (gPHA/gCOD)	0.28	0.39	0.34	0.26	0.40	0.27	0.30 ± 0.06

According to the COD, MLSS, and PHA results, feast/famine conditions were successfully established, and PHA-accumulating microorganisms were enriched in the SBR. The maximum PHA content (32.3% gPHA/gMLSS) was achieved in the 12^th^ cycle after 4 h of SBR operation. The sludge sample at that time point was collected for microbial community analysis using MiSeq and for investigation of PHA-accumulating microorganisms using FISH*.*

### Microbial community analysis using 16 S rRNA gene amplicon sequencing

**Figure 3:**
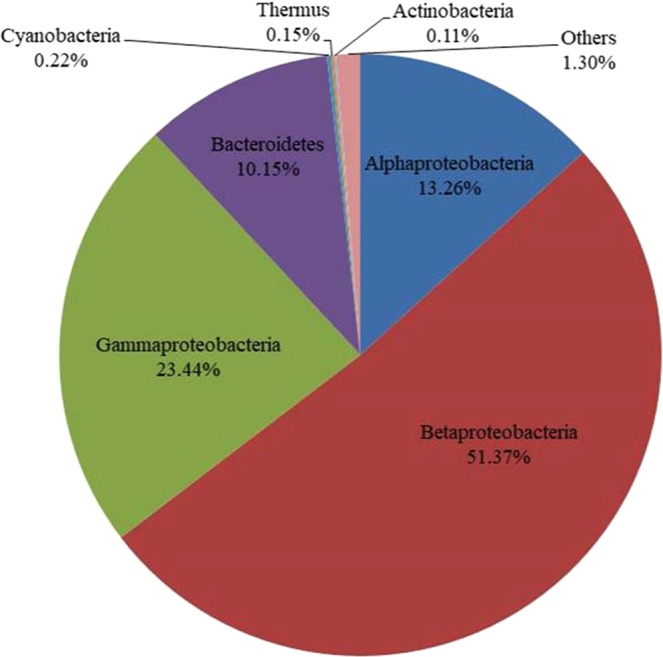
Microbial community analysis using 16S rRNA gene amplicon sequencing (MiSeq) in the sludge when the PHA content was at its maximum (12^th^ cycle of SBR operation), shown as percent relative abundances.

The microbial community in the sludge sample that achieved the maximum PHA content was analyzed using 16S rRNA gene amplicon sequencing (MiSeq). Table 3 shows the results. The relative abundances of different phyla are summarized as percentages in Fig. 3. Proteobacteria, including Betaproteobacteria (51.37%), Gammaproteobacteria (23.44%), and Alphaproteobacteria (13.26%), was the most abundant phylum. Several microorganisms in these three classes of Proteobacteria, such as *Cupriavidus necator*, *Burkholderia cepacia*, *Alcaligenes latus, Thauera selenatis*, *Plasticicumulans acidovorans*, and *Caulobacter crescentus*, are known PHA-accumulating microorganisms^1,9,28,29^. Bacteroidetes (10.15%) was also found in the sludge sample. No microorganisms in this phylum have been reported to accumulate PHA. However, Bacteroidetes species are likely to play a role in protein degradation in the system^30,31^, and they have been found in activated sludge systems^32,33^.

**Table 3:** Microbial community analysis using 16S rRNA gene amplicon sequencing (MiSeq) in the sludge when the PHA content was at its maximum (the 12^th^ cycle of SBR operation), presented as percent relative abundance values.

Phylum	Class	Order	Family	Genus
Proteobacteria (88.07%)	Betaproteobacteria (51.37%)	Rhodocyclales (51.07%)	Rhodocyclaceae (51.07%)	*Thauera* (48.88%)
				Unclassified (1.77%)
				Others (0.42%)
		Burkholderiales (0.30%)	Comamonadaceae (0.30%)	Unclassified (0.30%)
	Gammaproteobacteria (23.44%)	Alteromonadales (20.14%)	Chromatiaceae (20.14%)	Others (20.14%)
		Xanthomonadales (3.30%)	Xanthomonadaceae (3.30%)	*Aquimonas* (1.77%)
				*Luteimonas* (0.78%)
				Unclassified (0.75%)
	Alphaproteobacteria (13.26%)	Rhodobacterales (10.12%)	Rhodobacteraceae (6.29%)	Unclassified (4.55%)
				*Rhodobacter* (1.32%)
				*Rhodobaca* (0.25%)
				*Amaricoccus* (0.16%)
			Hyphomonadaceae (3.84%)	*Hyphomonas* (3.84%)
		Caulobacterales (2.21%)	Caulobacteraceae (2.21%)	Unclassified (2.21%)
		Rhizobiales (0.65%)	Hyphomicrobiaceae (0.37%)	*Devosia* (0.37%)
			Phyllobacteriaceae (0.28%)	Unclassified (0.28%)
		Sphingomonadales (0.27%)	Sphingomonadaceae (0.27%)	*Sphingopyxis* (0.27%)
Bacteroidetes (10.15%)	Cytophagia (4.5%)	Cytophagales (4.5%)	Cyclobacteriaceae (4.5%)	Unclassified (4.5%)
	Flavobacteriia (5.65%)	Flavobacteriales (5.65%)	Cryomorphaceae (3.95%)	Unclassified (2.40%)
				Others (1.55%)
			Flavobacteriaceae (1.70%)	*Flavobacterium* (1.70%)
Cyanobacteria (0.22%)	Chloroplast (0.22%)	Chlorophyta (0.22%)	Unclassified (0.22%)	Unclassified (0.22%)
Thermus (0.15%)	Deinococci (0.15%)	Deinococcales (0.15%)	Trueperaceae (0.15%)	*B-42* (0.15%)
Actinobacteria (0.11%)	Actinobacteria (0.11%)	Actinomycetales (0.11%)	Microbacteriaceae (0.11%)	Unclassified (0.11%)
Others (1.30%)

Table 4 shows the most abundant families and/or genera in the sludge. *Thauera* was determined to be the most abundant genus, with a relative abundance of 48.88%. *Thauera* is a genus in the family Rhodocyclaceae of the order Rhodocyclales of Betaproteobacteria. This organism has been reported to accumulate PHA in mixed microbial culture systems under feast/famine conditions^13,16,17,33,34^. Despite the different techniques in microbial community analysis, the results are in agreement with those of Lemos *et al*., who used FISH for microbial community analysis and found that *Thauera* was dominant at 49.4% and 41.1% of the total bacteria in SBRs fed with acetate under feast/famine conditions at SRTs of 1 d and 10 d, respectively^13^. Nevertheless, the presence of *Thauera* does not confirm its *in situ* capability to accumulate PHA in the system. Alphaproteobacteria, Betaproteobacteria, and Gammaproteobacteria were the dominant groups of microorganisms, and their PHA-accumulating capabilities have been previously reported. Therefore, *in situ* determination of the PHA-accumulating microorganisms was performed focusing on these groups, as discussed in the following section.

**Table 4:** The most abundant families and/or genera in the sludge when the PHA content was at its maximum (12^th^ cycle of SBR operation).

Family and/or genus (% relative abundance)	Class	Phylum
*Thauera* (48.88%)	Betaproteobacteria	Proteobacteria
Chromatiaceae (20.14%)	Gammaproteobacteria	
*Hyphomonas* (3.84%)	Alphaproteobacteria	
Caulobacteraceae (2.21%)		
*Aquimonas* (1.77%)		

### Investigation of *in situ* PHA-accumulating microorganisms by FISH

FISH was conducted to confirm the roles of Alphaproteobacteria, Betaproteobacteria, and Gammaproteobacteria in PHA accumulation. Based on the PHA accumulation results in the SBR reactor, the most abundant PHA-accumulating sludge was found 4 h into the 12^th^ cycle (32.2% gPHA/gMLSS), as shown in Fig. 2c. This sludge sample was subjected to both 16S rRNA gene amplicon sequencing and FISH analysis. Nile blue A (yellow) and DAPI (blue) were used to stain the PHA content and the chromosomes of all microorganisms, respectively, together with specific oligonucleotide probes labeled with Cy-3 dye (red) for Alphaproteobacteria (ALF1b+ALF968), Betaproteobacteria (BET42a), Gammaproteobacteria (GAM42a), and *Thauera* (Thau832).

**Figure 4:**
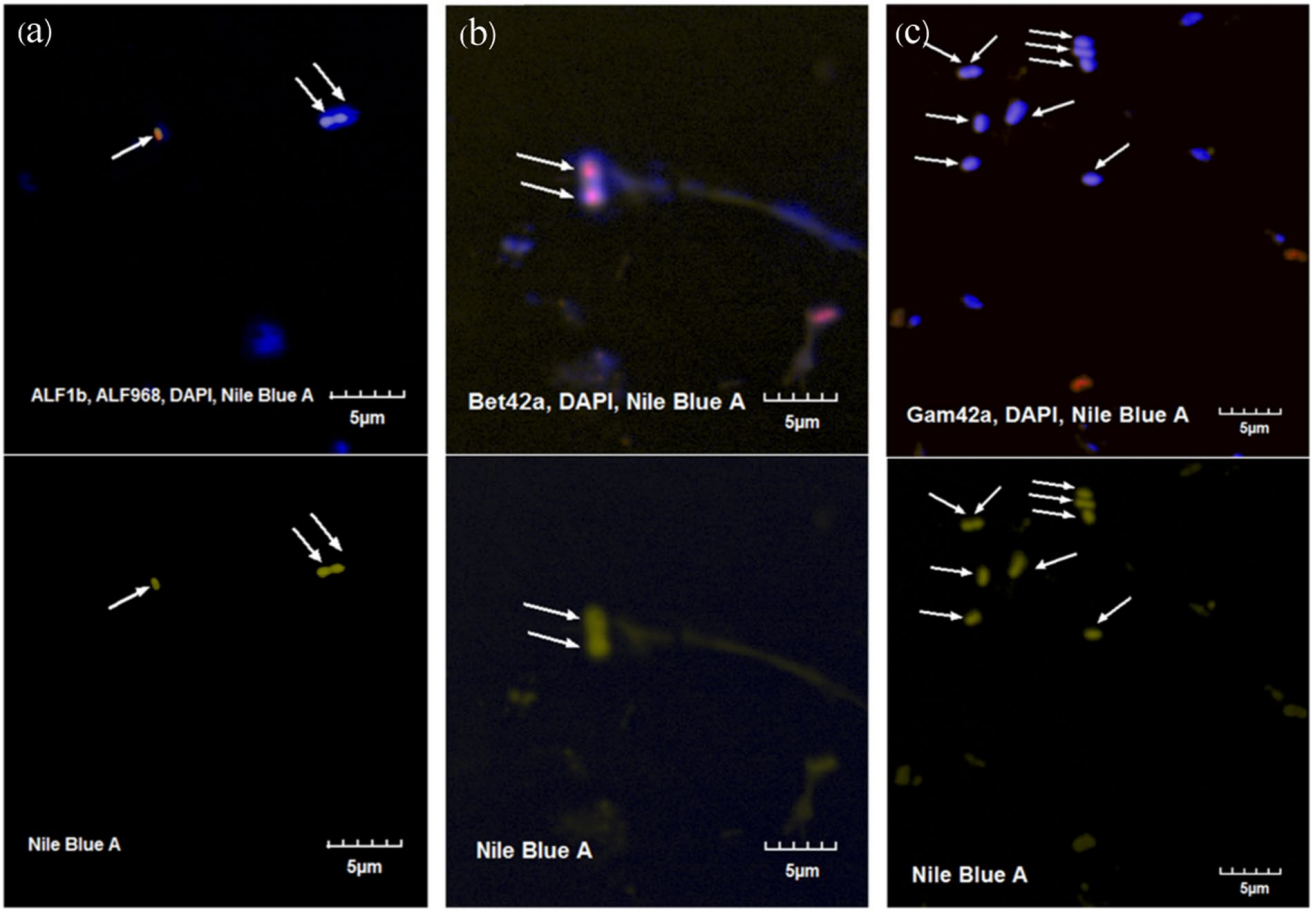
Fluorescence microscopy images of the mixed culture stained with DAPI (blue) for total microbial cells and Cy3-labeled probe (red) for Alphaproteobacteria (ALF1b+ALF968) (**a**), Betaproteobacteria (BET42a) (**b**) and Gammaproteobacteria (GAM42a) (**c**). The yellow color and arrow indicate the overlay of the Nile blue A stain identifying PHA accumulation in a particular cell.

Figure 4 shows examples of fluorescence microscopy images of the mixed culture. For each probe hybridization reaction, 20 images were taken to quantify the proportion of the specific bacterial groups in the total community. Alphaproteobacteria (ALF1b+ALF968), Betaproteobacteria (BET42a), and Gammaproteobacteria (GAM42a) were found at 21.6% (377 out of 1,744 cells), 38.7% (179 out of 462 cells) and 31.2% (113 out of 362 cells), respectively. These results suggest the predominance of Proteobacteria in the SBR reactor, in agreement with the results from 16S rRNA gene amplicon sequencing (MiSeq), which found that the relative abundance of Proteobacteria was up to 88% of the total sequences. Previous studies also observed a predominance of Proteobacteria in mixed PHA-producing microbial systems^16,17,34^. The proportion of Betaproteobacteria was slightly higher than those of Gammaproteobacteria and Alphaproteobacteria, as also determined from the MiSeq results. However, FISH is based on manual microscopic observation, and the number of detected cells was much lower than the MiSeq result; therefore, FISH was regarded as semiquantitative method.

Positive Nile blue A staining indicates PHA accumulation. Among the total PHA-accumulating bacteria under each probe hybridization condition, 15.1% (8 out of 53 cells) were associated with Alphaproteobacteria and 33.8% (24 out of 71 cells) with Betaproteobacteria. PHA-accumulating Gammaproteobacteria were not found among the 62 PHA-accumulating bacterial cells. This study indicated that some Alphaproteobacteria and Betaproteobacteria were responsible for PHA accumulation and that accumulation by Betaproteobacteria was the most significant. This study did not identify the role of Gammaproteobacteria in PHA accumulation. Our findings were in contrast with those of Johnson *et al*.^4^, who found that a Gammaproteobacterium was the dominant PHA-accumulating microorganism in an SBR fed with acetate operated at an SRT of 1 d. Notably, in our study we chose to use an SRT of 10 d. The differences in SRTs could have effects on microbial communities and PHA-accumulating microorganisms.

Based on our 16S rRNA gene amplicon sequencing analysis, Betaproteobacteria accounted for 51.4% of the total microorganisms in the reactor. *Thauera* (from betaproteobacterial class) was found at 48.88%. Therefore, we further investigated *Thauera* species using FISH and Nile blue A staining, as these bacteria might be the dominant strains accumulating PHA.

**Figure 5:**
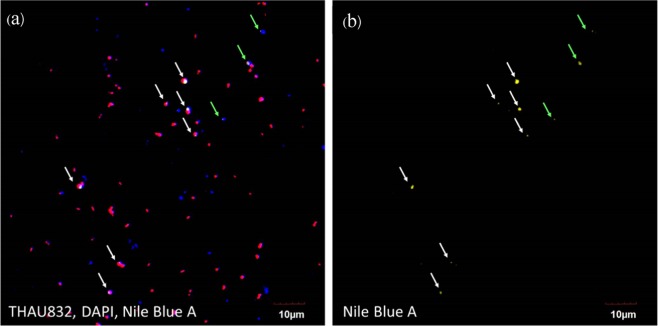
(**a**) Fluorescence microscopy image of the mixed culture stained with DAPI (blue) for total microbial cells, Cy3-labeled probe (red) for *Thauera* spp. (THAU832), and Nile blue A (yellow) for PHA accumulation. (**b**) Fluorescence microscopy image of Nile blue A (yellow) staining for PHA accumulation. White arrows indicate PHA-accumulating *Thauera* spp., and green arrows indicate PHA accumulation by other microorganisms.

Probe THAU832^35,36^ was incorporated to investigate the role of *Thauera* spp. on PHA accumulation as shown in Fig. 5. This probe was selected because it is capable of detecting 91.3% (379 in total 415 sequences) of *Thauera* species in the SILVA SSU r132 ribosomal RNA database (TestProbe achieved on September 15, 2019)^37^. This probe could negligibly bind to other groups (i.e., 3 sequences of uncultured *Rhodocyclaceae*, 2 *Longilinea*, 2 *Zoogloea*, 1 uncultured *Deferribacteraceae*, 1 SM1A02 *Phycisphaeraceae*, 1 *Azoarcus*, 1 uncultured *Xanthomonadaceae*, and 1 *Aminivibrio*). Of 176 Nile blue A-stained PHA cells in 20 microscopic fields, 140 cells were bound to the probe THAU832, accounting for 80.3%. This study clearly indicated the ability of *Thauera* spp. for PHA accumulation in feast/famine mixed-culture SBR feeding with acetate as a main carbon source.

Other studies have also found *Thauera* to accumulate PHA in mixed microbial cultures under feast/famine feeding conditions^13,16,34^. Lemos *et al*., who used FISH for microbial community analysis, found that *Thauera* was dominant and responsible for PHA accumulation in the system^13^. Jiang *et al*. also reported the predominance of *Thauera selenetis* and *Plasticicumulans acidivorans* in an SBR system fed with a mixture of acetate and lactate under feast/famine conditions at an SRT of 1 d^9^. However, Jiang *et al*. suggested that *Thauera* sp. was a specialist at lactate conversion, while *Plasticicumulans acidivorans* was a specialist at acetate conversion^9^. Ciggin *et al*. also found that *Thauera* and *Azoarcus* were dominant and capable of PHA accumulation in an SBR system fed with acetate under feast/famine conditions at an SRT of 2 d^34^. Moita and Lemos reported that *Thauera*, *Amaricoccus*, and *Zoogloea* were the dominant microorganisms in an SBR system fed with bio-oil from fast pyrolysis under feast/famine conditions^16^. In addition, a recent study by Huang *et al*. found that *Thauera* was a predominant microorganism in mixed microbial cultures producing PHA, which were fed with mixtures of protein, carbohydrate, and volatile fatty acids, using 16S rRNA gene amplicon sequencing (HiSeq) although the role and contribution of this microorganism to PHA accumulation was not confirmed^7^.

Despite the large variety of operating conditions, seed sludges, and environmental factors, *Thauera* has been found in many PHA-accumulating mixed microbial cultures under feast/famine conditions, suggesting that it is a strong competitor and appears to play important roles in PHA accumulation under feast/famine conditions.

In this study, we applied two complementary techniques, namely, 16S rRNA gene amplicon sequencing (MiSeq) and FISH with fluorescence dye-based PHA staining, to systematically identify *in situ* PHA-accumulating microorganisms in mixed microbial cultures. Further studies should be designed to investigate methods to promote PHA accumulation by *Thauera* spp.

## Conclusions

The mixed microbial culture enriched with acetate in the SBR under feast/famine feeding conditions was able to accumulate a maximum PHA content of 32.3% (gPHA/gMLSS). The results from 16S rRNA gene amplicon sequencing analysis (MiSeq) show that the dominant group of microorganisms in the sludge with the maximum PHA content was Proteobacteria, consisting of Alphaproteobacteria (13.26% of the total sequences), Betaproteobacteria (51.37% of the total sequences), and Gammaproteobacteria (23.44% of the total sequences). *Thauera* (Betaproteobacteria), a known PHA-accumulating microorganism, was found at a high relative abundance of 48.88% of the total sequences. FISH analysis with fluorescence PHA staining revealed that microorganisms belonging to Alphaproteobacteria and Betaproteobacteria in the sludge were capable of accumulating PHA. *Thauera* spp. from Betaproteobacteria contributed 80.3% of the total PHA-accumulating cells.
